# Effects of ulinastatin therapy in emergency severe multiple trauma: A single-center randomized controlled trial

**DOI:** 10.1097/MD.0000000000032905

**Published:** 2023-02-17

**Authors:** Haiting Xu, Wei Jiao, Yunfei Zhang, Xiaoyan Deng, Rongrong Dai, Lei Chen

**Affiliations:** a Department of Emergency, The 904^th^ Hospital of Joint Logistic Support Force, Wuxi, China; b Department of Nursing, The 904^th^ Hospital of Joint Logistic Support Force, Wuxi, China; c Department of Neurosurgery, The 904^th^ Hospital of Joint Logistic Support Force, Wuxi, China.

**Keywords:** 30-day mortality, outcome, RCT, severe multiple traumas, ulinastatin

## Abstract

Severe multiple traumas are one of the most common diseases and carry a significant financial burden with high disability and mortality. There are no effective drugs in the clinical management of severe multiple traumas, and there is an absence of evidence-based medicine concerning the treatment of severe multiple traumas.

**Methods:**

The present study explored whether ulinastatin (UTI) can improve the outcome of severe multiple traumas. The present research included patients who were hospitalized in intensive care units after being diagnosed with severe multiple trauma. Patients received UTIs (400,000 IU) or placebos utilizing computer-based random sequencing (in a 1:1 ratio). The primary outcome measures were 30-day mortality, multiple organ dysfunction syndrome, inflammatory response, coagulation function, infection, liver function, renal function, and drug-related adverse effects.

**Results:**

A total of 239 individuals were classified into 2 groups, namely, the placebo group (n = 120) and the UTI group (n = 119). There were no statistically significant differences in baseline clinical data between the 2 groups. The 30-day mortality and multiple organ dysfunction syndrome in the UTI group were remarkably improved compared with those in the placebo group. UTI can protect against hyperinflammation and improve coagulation dysfunction, infection, liver function, and renal function. UTI patients had markedly decreased hospitalization expenditures compared with the placebo group.

**Conclusion:**

The findings from the present research indicated that UTIs can improve the clinical outcomes of patients with severe multiple traumas and have fewer adverse reactions.

## 1. Introduction

Injuries caused by trauma remain one of the most common causes of death in the world, especially emergency severe multiple traumas combined with traumatic brain injury.^[1,2]^ With the development of orthopedics, neurosurgery, and critical care medicine, the complications of multiple traumas combined with traumatic brain injury have decreased significantly, while the mortality rate is still high, which restricts the improvement of the prognosis of patients.^[3]^ Severe traumatic stress can promote systemic inflammatory reactions, and posttraumatic hyperinflammation can cause coagulation dysfunction, which leaves the patient vulnerable. An imbalance and high intensity of systematic inflammatory response syndrome and compensatory anti-inflammatory response syndrome trigger disease progression, and multiple organ dysfunction syndrome (MODS) ultimately affects the patient’s long-term prognosis.^[2]^ How to effectively control posttraumatic hyperinflammation after severe multiple traumas and how to prevent and alleviate MODS play a very important role in early-time treatments.

Ulinastatin (UTI) is a serine protease inhibitor with a molecular weight of 67,000 that is purified from human urine. The primary pharmacological activities of this compound were anti-inflammatory, immunoregulatory, and organ protection.^[4–6]^ Drugs act as anti-inflammatory and antiapoptotic agents in the treatment of acute inflammatory disorders, such as sepsis and ischemia–reperfusion injury.^[7]^ He et al reported that cardiological surgery patients may benefit from UTIs by inhibiting postoperative inflammatory processes and providing pulmonary protection through a meta-analysis including 15 randomized controlled trials.^[8]^ Our recent animal studies also found that UTI can alleviate traumatic brain injury by inhibiting oxidative stress and apoptosis,^[5]^ improve early brain injury after intracerebral hemorrhage by inhibiting oxidative stress and neuroinflammation via the ROS/MAPK/Nrf2 signaling pathway,^[9]^ and relieve cerebral ischemia–reperfusion injury.^[10]^ UTIs have been widely used in China for the treatment of patients with inflammatory disorders, postoperative organ protection, and shock.^[11]^ A recent study showed that Xubijing combined with UTI has a good effect on the treatment of patients with traumatic sepsis and can also significantly improve patients’ conditions by reducing inflammation, improving immune function, and promoting liver function recovery.^[12]^ A meta-analysis of randomized controlled trials also indicated that UTI seemed to show a beneficial effect for acute respiratory distress syndrome patient treatment but lacked a larger sample size of randomized controlled trials.^[13]^

However, because of the unique nature of emergency severe multiple trauma patients and the lack of evidence-based medical studies with large sample sizes, the efficacy of UTI treatment remains unclear. Thus, the present study aimed to test the hypothesis that the administration of UTI would improve the outcome and alleviate posttraumatic hyperinflammation after severe multiple traumas.

## 2. Methods

### 2.1. Study design

**Figure 1.**
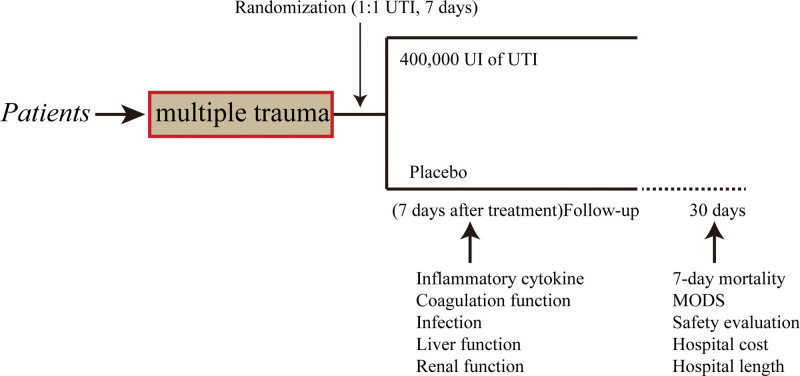
Study design.

A placebo-controlled, parallel-arm, randomized experiment was carried out in Jiangsu from January 2019 to December 2021. A total of 267 patients were screened over this period, and 239 of these were initially enrolled in the study to form the intention-to-treat population. To determine if the intervention is superior, the present research was conducted. The Clinical Research Ethics Committees of the 904^th^ Hospital of PLA endorsed the methodology used in the present research (2019-YXLL-101) and followed the Declaration of Helsinki. It was registered with the registration number CWXH-IPR-2018004 (date: January 20, 2018). The protocol for the research was subjected to approval granted by the Ethics Committees of all the collaborating centers. Those patients whose competence could be demonstrated by their comprehensive awareness of time, place, and person, along with their comprehension of the investigator’s explanation of the trial, were asked to obtain written informed consent for the study. In addition, patients were allocated at random (1:1) and were administered an intravenous infusion of either 200,000 IU UTI (Guangdong Tianpu Biochemical Pharmaceutical Co., Ltd., Guangzhou, China, H20040506, 2 mL:100,000 IU) or placebos dissolved in 250 mL of 0.9% saline given intravenously over 1 hour every 12 hours for 7 days. For patients with fluid restriction, 100 mL of 0.9% saline could be used.^[6]^ Infusion could be interrupted for 1 day if there was a >3-fold increase in liver enzymes over baseline levels following the procedure (Fig. 1). The last checkup was performed 30 days following the procedure.

### 2.2. Patients enrolled in the study and sample selection procedures

Patients were included in the present research in the emergency intensive care unit. The following were the criteria for inclusion: patients aged 25 to 70 years; >2 anatomical sites injured and injury severity score ≥ 16; time from injury to hospitalization <12 hours; estimated posttraumatic survival time >72 hours; and randomly assigned to receive either UTI or a placebo. The exclusion criteria were as follows: patients who were unlikely to be salvaged upon admission; allergic to UTI; received anticoagulant medication within 48 hours before hospitalization; pregnant women and patients with malignant tumors; treatment with immunosuppressive drugs; multiple organ dysfunction; and other explanations were discovered by researchers.

### 2.3. Randomization and concealment

With the aid of SPSS software (version: 14.0) (SPSS Institute, Anhui Medical University, Hefei, China), permuted-block randomization was carried out based on a computer system that used an allotment list to produce random numbers (in a 1-to-1 ratio). This was carried out by a statistician who was not a member of the research team to maintain the integrity and blinding of the research. The outcomes of the random sampling process were enclosed in prenumbered envelopes and kept at the location of the research until the study’s conclusion was reached. The study medicines were delivered by a research nurse following the random assignment sequence. Both the research participants and the patients were unaware of which medicine was being applied in the trial. In the event of an emergency, such as acute hepatic failure, 2 experts might recommend that the treatment allotment be unmasked and that the study medicine be adjusted or discontinued if needed, according to the protocol. All of the occurrences were recorded in detail. Then, we acquired information on the patient’s demographics, medical histories, and pertinent investigation findings.

### 2.4. Outcome assessment

All clinical and imaging data and treatment were subjected to assessment by a masked independent diagnostic and assessment committee. This committee included 2 researchers who were trained before the start of the present research and did not engage in the clinical care of patients. The primary endpoint of this study was 30-day mortality. The secondary endpoints included the incidence of MODS; inflammatory cytokine levels (pretherapy and posttreatment), such as serum tumor necrosis factor-α and interleukin-6; coagulation function, such as the plasma prothrombin time, activated partial thromboplastin time and fibrinogen (FIB) levels, was measured using an automatic coagulation analyzer; serum infection index levels, such as white blood cell count, C-reactive protein, and procalcitonin; liver function index levels, such as alanine aminotransferase and aspartate aminotransferase; and renal function index levels, such as creatinine and urea nitrogen.

### 2.5. Safety evaluation and complications

We kept track of the length of time spent in the intensive care unit, and the most prevalent adverse effects of UTIs included granulocytopenia and abnormal liver enzymes. Other rare complications include diarrhea, vomiting, and allergies. All complications were confirmed and recorded by physical examination after 2 doctors and nurses; granulocytopenia was diagnosed by routine blood level detection. Abnormal liver enzymes were diagnosed by liver function tests. Finally, we check the related index every 2 days over the first 14 days.

### 2.6. Postoperative hospital stays and hospitalization costs

As previously reported, severe multiple traumas or critical care patients have remarkably longer hospital stays and higher hospitalization expenses. Thus, the present research examined the difference in the overall duration of hospital stay and healthcare costs among patients belonging to the 2 groups.

### 2.7. Sample size estimates

In a previous study,^[6]^ the primary endpoint showed that the 28-day mortality in the UTI group was 7.3% and 20.3% in the placebo group. The sample size was calculated according to an *α* of 0.05 and a statistical power of 80%, and 236 patients were enrolled (118 in each category). We decided to enroll 240 patients (120 in each category). Ultimately, we enrolled 120 patients in the UTI group and 119 patients in the placebo group. The study database included all baselines, and outcome data were entered by a study nurse.

### 2.8. Statistical analysis

Data from the baseline as well as outcome assessments were input into the database by a research nurse. The information was gathered on handwritten forms and stored in a digital database that was password secured. All continuous variables are presented as the mean ± SD. SPSS 19.0 statistical software (SPSS, Inc., Chicago, IL, USA) was used for the statistical analyses. Measurement data with a nonnormal distribution are represented by M (Q1, Q3). Independent-samples *t* tests were used to assess quantitative data. Qualitative data were compared with the chi-square test or Fisher exact *t* test. A value of *P* < .05 was considered statistically significant.

## 3. Results

**Figure 2.**
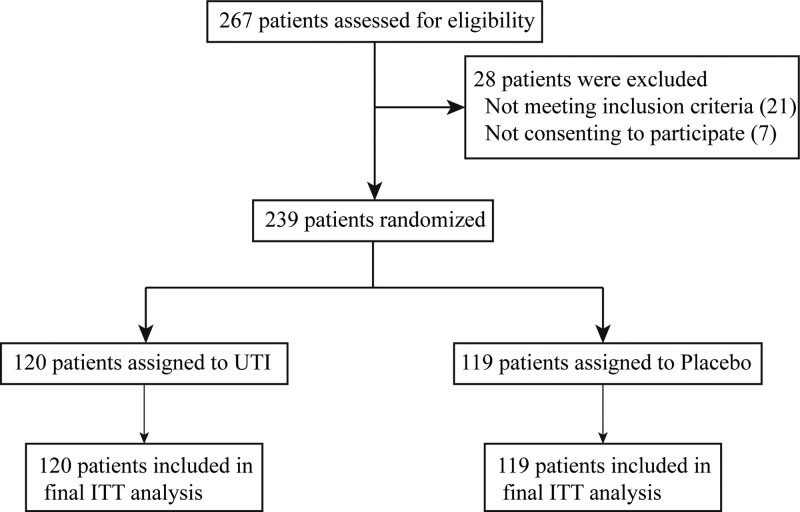
Trial profile.

A total of 267 patients were evaluated between January 2019 and December 2021, and 239 of these were initially enrolled in the study to form the intention-to-treat population, which was given UTI (n = 120) or placebo (n = 119) treatment in a random manner. There were no cases of open blindness observed throughout the research period. Furthermore, no statistically significant differences were discovered in terms of the baseline data between the 2 subgroups (Table 1). None of the patients were lost to follow-up throughout the present research. The eventual intention-to-treat analysis incorporated all of the patients (Fig. 2). The concluding appointment with the last randomly selected patient took place on March 15, 2022.

**Table 1:** Comparison of baseline data.

	UTI group (n = 91)	Placebo group (n = 90)	*P*
Age (yr, mean ± SD)	52.7 ± 3.8	53.5 ± 4.1	.175
Gender, no. (%)			.705
Male	67 (73.63%)	64 (71.11%)	
Female	24 (26.37%)	26 (28.88%)	
BMI (kg/cm^2^, mean ± SD)	22.6 ± 2.1	23.1 ± 2.5	.147
ISS score	28.6 ± 9.5	27.9 ± 9.1	.613
Cause of disease, no. (%)			.582
Biliary	61 (67.03%)	57 (63.33%)	
Hyperlipidemic	18 (19.78%)	19 (21.11%)	
Alcoholic	12 (13.09%)	14 (15.56%)	
Smoking history, no. (%)			.483
Yes	53 (%)	57 (%)	
No	38 (%)	33 (%)	
Drinking history, no. (%)			.371
Yes	58 (58.24%)	63 (70.00%)	
No	33 (41.76%)	27 (30.00%)	
Living environment, no. (%)			.604
Town	59 (64.84%)	55 (61.11%)	
Countryside	32 (35.16%)	35 (38.89%)	
Past medical history, no. (%)			
Hypertension	31 (34.07%)	29 (32.22%)	.792
Hyperlipidemia	35 (38.46%)	37 (41.11%)	.716
Diabetes	28 (30.77%)	29 (32.22%)	.833
Cholelithiasis	17 (18.68%)	16 (17.78%)	.875
Previous pancreatitis, no. (%)	9 (9.89%)	10 (11.11%)	.789

BMI = body mass index, ISS = injury severity score, SD = standard deviation, UTI = ulinastatin.

### 3.1. The primary endpoint and risk of MODS

After treatment, the total 30-day mortality was 16.74% (40/239), according to the findings. The 30-day mortality was 15.38% (14/120) in the UTI group and 28.89% (26/119) in the placebo control group. As opposed to the UTI group, the placebo group exhibited a remarkably greater 30-day mortality rate, with a significant difference (*P* = .035, Table 2). For the MODS incidence rate, UTI was associated with a lower total MODS incidence rate versus the placebo control group (13.33% vs 34.07%, *P* = .031, Table 2).

**Table 2:** Comparison of 7-day mortality and clinical efficacy.

	UTI group (n = 120)	Placebo group (n = 119)	*P*
30-d mortality, no. (%)	14 (15.38%)	26 (28.89%)	.035
MODS, no. (%)	16 (13.33%)	31 (34.07%)	.031

MODS = multiple organ dysfunction syndrome, UTI = ulinastatin.

### 3.2. The secondary endpoints

Before UTI or placebo treatment, there was no significant difference in the inflammatory cytokine levels, coagulation function, serum infection index levels, liver function index levels, or renal function index levels between the 2 groups (*P* > .05). After UTI treatment, the inflammatory cytokine levels, coagulation function, infection index levels, liver function index levels, and renal function index levels improved significantly in the UTI group compared with the placebo group (*P* < .05, Table 3).

**Table 3:** Comparison of the secondary end-points.

	Before treatment			After treatment		
	UTI (n = 120)	Placebo (n = 119)	*P*	UTI (n = 120)	Placebo (n = 119)	*P*
Inflammatory cytokine, mean ± SD						
TNF-α (pg/mL)	46.39 ± 9.27	45.81 ± 8.77	.620	26.23 ± 5.14	30.19 ± 5.70	<.001
IL-6 (pg/mL)	25.43 ± 4.12	26.16 ± 3.80	.156	15.55 ± 3.14	19.27 ± 3.53	<.001
Coagulation function, mean ± SD						
PT (s)	22.19 ± 3.23	21.62 ± 3.02	.160	11.25 ± 2.47	14.38 ± 2.91	<.001
APTT (s)	36.19 ± 4.08	35.27 ± 3.91	.076	29.07 ± 3.51	33.59 ± 3.84	<.001
D-D (mg/L)	4.95 ± 1.26	4.83 ± 1.41	.488	2.15 ± 0.84	3.11 ± 0.96	<.001
FIB (g/L)	1.13 ± 0.24	1.17 ± 0.27	.227	3.15 ± 0.52	3.43 ± 0.64	<.001
Infection index levels, mean ± SD						
CRP (mg/L)	30.15 ± 4.07	29.83 ± 3.91	.536	4.82 ± 0.86	6.51 ± 1.10	<.001
PCT (ng/L)	2.39 ± 0.64	2.25 ± 0.58	.078	0.69 ± 0.18	0.75 ± 0.27	.044
WBC (×10^9^/L)	16.25 ± 7.92	17.13 ± 8.48	.408	9.84 ± 5.11	10.71 ± 5.92	.225
Liver function index levels, mean ± SD						
ALT (U/L)	33.29 ± 3.65	32.79 ± 3.41	.275	50.18 ± 4.51	52.91 ± 4.47	<.001
AST (U/L)	32.29 ± 2.95	33.01 ± 3.09	.067	45.33 ± 3.65	48.37 ± 4.08	<.001
Renal function index levels, mean ± SD						
SCr	78.35 ± 8.92	79.93 ± 9.47	.186	88.76 ± 10.71	101.52 ± 11.87	<.001
BUN	5.09 ± 1.81	5.22 ± 1.94	.593	9.33 ± 3.01	11.02 ± 3.41	<.001

ALT = alanine aminotransferase, APTT = activated partial thromboplastin time, AST = aspartate aminotransferase, BUN = urea nitrogen, CRP = C-reactive protein, D-D = d-dimer, FIB = fibrinogen, IL-6 = interleukin-6, PCT = procalcitonin, PT = prothrombin time, SCr = serum creatinine, SD = standard deviation, TNF-α = tumor necrosis factor-α, UTI = ulinastatin, WBC = white blood cell.

### 3.3. Safety evaluation

The most prevalent adverse effects of UTI include granulocytopenia and abnormal liver enzymes. We found that 10 (8.33%) patients experienced granulocytopenia in the UTI group, while 6 (5.04%) patients experienced granulocytopenia in the placebo group, with no significant difference between the 2 groups. There were 21 (17.65%) cases of abnormal liver enzymes in the control group, while there were 39 (32.50%) cases of abnormal liver enzymes in the UTI group, which was significantly higher than the placebo group (*P* = .008, Table 4). Furthermore, no significant difference was identified between the 2 groups in the possible side effects of UTI-induced diarrhea (25.83% vs 16.81%, *P* = .089, Table 4) or vomiting (20.00% vs 12.61%, *P* = .122, Table 4). The incidence of allergies was higher in the UTI group than in the placebo group (17.50% vs 8.40%, *P* = .036, Table 4).

**Table 4:** Comparison of safety evaluation, and postoperative hospital stays and costs.

	UTI (n = 120)	Placebo (n = 119)	*P*
Granulocytopenia, no. (%)	10 (8.33%)	6 (5.04%)	.309
Abnormal liver enzymes, no. (%)	39 (32.50%)	21 (17.65%)	.008
Diarrhea, no. (%)	31(25.83%)	20 (16.81%)	.089
Vomiting, no. (%)	24 (20.00%)	15 (12.61%)	.122
Allergies, no. (%)	21 (17.50%)	10 (8.40%)	.036
Hospitalization stays, d, mean ± SD	28.64 ± 8.91	29.41 ± 9.17	.511
Hospitalization costs, CNY*10^4^, mean ± SD	8.15 ± 2.14	8.92 ± 2.48	.011

CNY = Chinese Yuan, SD = standard deviation, UTI = ulinastatin.

### 3.4. Postoperative hospital stays and hospitalization costs

In the UTI group, the average duration of stay was 28.64 days, whereas the value for the placebo group was 29.41 days, with no statistically significant difference (*P* = .511). The mean hospitalization expenditure of the UTI group was 81500 RMB, which was much less than the placebo control group’s cost of 89200 RMB (*P* = .011, Table 4).

## 4. Discussion

According to the findings of the current investigation, UTI may greatly reduce the incidence of total 30-day mortality and MODS in emergency severe multiple trauma patients. Additionally, we also found that UTI can protect against hyperinflammation and improve coagulation dysfunction, infection, liver function, and renal function. It also did not significantly increase the incidence rate of severe adverse effects other than allergies and abnormal liver enzymes, and this should be considered because the synergistic effect after the combined application of other drugs in some patients may lead to an increase in the proportion of patients with allergies. Moreover, it can also decrease the expenditure on hospitalization.

According to the findings, the total 30-day mortality was 16.74%, which was consistent with earlier reports.^[1]^ More than 5 million deaths occur each year due to trauma, making it one of the leading causes of death in the world.^[14]^ Motor vehicle accidents remain the most common cause of multiple traumas, especially for extraordinary amounts of severe multiple traumas.^[15,16]^ With the development of medical technology and the improvement of critical care management in the last 30 years, the overall mortality following multiple traumas has decreased from over 40% to 15%.^[16–18]^ While an individual survives major trauma, they may suffer psychological distress for the rest of their lives and/or suffer physical impairment that often leads to disability from work.^[16,19]^ Recent studies have found that severe multiple trauma patients usually die from multiorgan failure/multiorgan dysfunction syndrome (MOF/MODS), and the incidence rate of MOF is as high as 35 to 54%.^[16,20]^ It is vital to prevent and intervene with MOF/MODS. The management of severe multiple traumas requires intensive care management, preventing infection and identifying infection, nutritional support, and therapeutic agents.^[1,21–24]^ Additionally, to achieve further advancements, drugs need to be developed and clinically tested to target disease mechanisms. Unfortunately, no drug has been identified that consistently modulates the outcome of severe multiple traumas according to our data.

UTI is a 67 kDa glycoprotein purified from the urine of healthy humans that is a nonspecific protease inhibitor and a urinary trypsin inhibitor that is used to treat acute inflammatory disorders, sepsis, toxic shock, and hemorrhagic shock.^[6,25]^ Previous animal studies have also indicated that UTI can regulate inflammation, oxidative stress, apoptosis, immune regulation, and organ protection.^[4,26]^ In lipopolysaccharide-induced pulmonary injury models, UTI can alleviate pulmonary tissue injury via immunoregulation and ameliorate excessive inflammatory responses.^[27]^ Karnad^[6]^ reported that intravenous administration of UTIs can decrease the mortality of severe sepsis patients in a modified intention-to-treat analysis via a multicenter randomized controlled study. Park^[28]^ demonstrated that 300,000 IU of UTI can decrease the serum polymorphonuclear leukocyte elastase levels in trauma patients with hemorrhagic shock at 48 hours, while whether it can improve the outcomes is unclear. In the present study, UTI intervention was used to reduce the incidence rate of total 30-day mortality and MODS. Wei^[29]^ reported that an insulin pump combined with UTI can shorten the recovery time of clinical symptoms and reduce the levels of inflammatory factors after diabetic ketoacidosis complicated with pancreatitis. Xubijing combined with UTI can improve the outcome, decrease the inflammatory response, and promote the recovery of the patient’s immune function and liver function in traumatic brain injury.^[12,30]^ While almost all UTI research has focused on traumatic brain injury alone, there is a lack of evidence-based medicine for multiple traumas. Hence, the present study first approved a large sample single-center randomized controlled trial to confirm the clinical value of UTIs in severe multiple traumas. In the present study, we found that UTI can improve inflammation, coagulation function, infection, liver function, and renal function. Interestingly, FIB levels post-drug administration were 3.15 g/L and 3.4 g/L in the placebo group, and the difference may not have biological significance. We hypothesize that UTI can maintain FIB homeostasis, which could be a future work that needs to be carried out. We also found that it is necessary to pay attention to the prevention and treatment of allergic reactions and abnormal liver enzymes after UTI administration. The following were some of the study’s limitations. The results may not be generalized because this study was a single-center randomized controlled trial. In the present research, UTI was administered at a single dosage. In the future, more multicenter randomized controlled trials are needed to explore the clinical outcome of multiple traumas with varied dosages.

## 5. Conclusion

The findings of the present research suggest that UTI treatment may help to minimize the risk of mortality and MODS against hyperinflammation and improve coagulation dysfunction, infection, liver function, and renal function following severe multiple traumas. Furthermore, it resulted in a considerable reduction in hospitalization expenditures. To completely grasp the prospective application of UTIs in multiple trauma patients, more studies with individuals who receive varied dosages are needed.

## Acknowledgments

We hereby express our gratitude to Jiangsu Brilliant Biological Technology Co., Ltd. for providing technical and linguistic help.

## Author contributions

**Conceptualization:** Haiting Xu, Xiaoyan Deng, Lei Chen.

**Data curation:** Wei Jiao, Xiaoyan Deng.

**Formal analysis:** Wei Jiao, Yunfei Zhang, Xiaoyan Deng, Lei Chen.

**Investigation:** Haiting Xu, Yunfei Zhang, Rongrong Dai.

**Methodology:** Haiting Xu, Wei Jiao, Lei Chen.

**Resources:** Wei Jiao, Yunfei Zhang, Xiaoyan Deng, Rongrong Dai.

**Software:** Haiting Xu, Xiaoyan Deng.

**Supervision:** Wei Jiao, Rongrong Dai.

**Validation:** Haiting Xu, Wei Jiao, Yunfei Zhang, Lei Chen.

**Visualization:** Haiting Xu, Wei Jiao, Yunfei Zhang, Xiaoyan Deng, Rongrong Dai, Lei Chen.

**Writing – original draft:** Haiting Xu, Wei Jiao, Yunfei Zhang, Lei Chen.

**Writing – review & editing:** Xiaoyan Deng, Rongrong Dai, Lei Chen.
